# Distinct Circle of Willis anatomical configurations in healthy preterm born adults: a 3D time-of-flight magnetic resonance angiography study

**DOI:** 10.1186/s12880-025-01562-y

**Published:** 2025-01-30

**Authors:** Julien Greggio, Christina Malamateniou, Kelly Pegoretti Baruteau, Constantino Carlos Reyes-Aldasoro, Odaro J. Huckstep, Jane M. Francis, Wilby Williamson, Paul Leeson, Adam J. Lewandowski, Winok Lapidaire

**Affiliations:** 1 Department of Midwifery & Radiography, City St George’s, School of Health & Psychological Sciences, University of London, Clerkenwell Campus, London, EC1V 0HB UK; 2 Department of Neuroimaging, Kings’ College London, London, SE5 8AF UK; 3 Lysholm Department of Neuroradiology, National Hospital for Neurology and Neurosurgery, University College London Hospitals NHS Foundation Trust, London, WC1N 3BG UK; 4 Elizabeth Garrett Anderson, Institute for Women’s Health, University College London, London, UK; 5 Department of Computer Science, City St George’s, School of Health & Medical Sciences, University of London, London, EC1V 0HB UK; 6 Integrated Pathology Unit, Division of Molecular Pathology, The Institute of Cancer Research, Sutton, UK; 7 Department of Biology, United States Air Force Academy, Colorado Springs, CO USA; 8 MyCardium AI, Liverpool, UK; 9 School of Medicine, Trinity College Dublin, Dublin, Ireland; 10 Oxford Cardiovascular Clinical Research Facility, Division of Cardiovascular Medicine, Radcliffe Department of Medicine, Level 1, Oxford Heart Centre, John Radcliffe Hospital, University of Oxford, Oxford, OX3 9DU UK; 11 Nuffield Department of Population Health, University of Oxford, Oxford, OX3 7LF UK

**Keywords:** Preterm birth, Circle of Willis, Anatomical variations, Magnetic resonance angiography, Vascular remodelling

## Abstract

**Background:**

Preterm birth (< 37 weeks’ gestation) alters cerebrovascular development due to the premature transition from a foetal to postnatal circulatory system, with potential implications for future cerebrovascular health. This study aims to explore potential differences in the Circle of Willis (CoW), a key arterial ring that perfuses the brain, of healthy adults born preterm.

**Methods:**

A total of 255 participants (108 preterm, 147 full-term) were included in the analysis. High-resolution three-dimensional Time-of-Flight Magnetic Resonance Angiography (3D TOF MRA) datasets were analysed, measuring vessel diameters and classifying segments into different groups of CoW anatomical variations. Statistical comparisons assessed the prevalence of each variant group between preterm and full-term populations, as well as the relationship between CoW variability, sex, and degree of prematurity.

**Results:**

We identified 164 participants with variant CoW configurations. Unilateral segment hypoplasia (30%) and unilateral segment absence (29%) were the most common variations, with over 50% related to the posterior communicating artery (PComA). However, the incidence of absent segments was lower in preterm adults, who were more likely to exhibit variants associated with complete CoW configurations compared to full-term adults (*p* = 0.025). Preterm males had a higher probability of a group 1 variant (circles with one or more hypoplastic segments only) than the full-term group (*p* = 0.024). In contrast, preterm females showed higher odds of a group 4a variant (circles with one or more accessory segments, without any absent segments) in comparison to their full-term counterparts (*p* = 0.020).

**Conclusions:**

Preterm birth is linked to a distinct vascular phenotype of CoW in adults born preterm, with a higher likelihood of a CoW configuration with hypoplastic segments but a lower likelihood of absent segments. Future work should focus on larger prospective studies and explore the implications of these findings for normal development and cerebrovascular disease. Furthermore, TOF MRA might be a useful adjunct in the neurovascular assessment of preterm-born individuals.

**Supplementary Information:**

The online version contains supplementary material available at 10.1186/s12880-025-01562-y.

## Background

Preterm birth, which is defined as birth at less than 37 weeks’ gestation, is associated with systemic alterations in vascular structure and function [1]. It is also known to alter normal brain growth, resulting in changes in cortical maturation and structural modifications in deep grey matter regions and the cerebral vasculature [2–4], including distinctive cerebral arterial patterns [5]. These alterations can lead to a reduced cortical surface area, decreased vascular tortuosity, and modified white matter properties [5–7]. The altered neurodevelopment patterns commonly observed in preterm infants may persist into adulthood [4]. Furthermore, preterm populations may experience higher incidence of cardiovascular disease and stroke [8, 9].

**Fig. 1:**
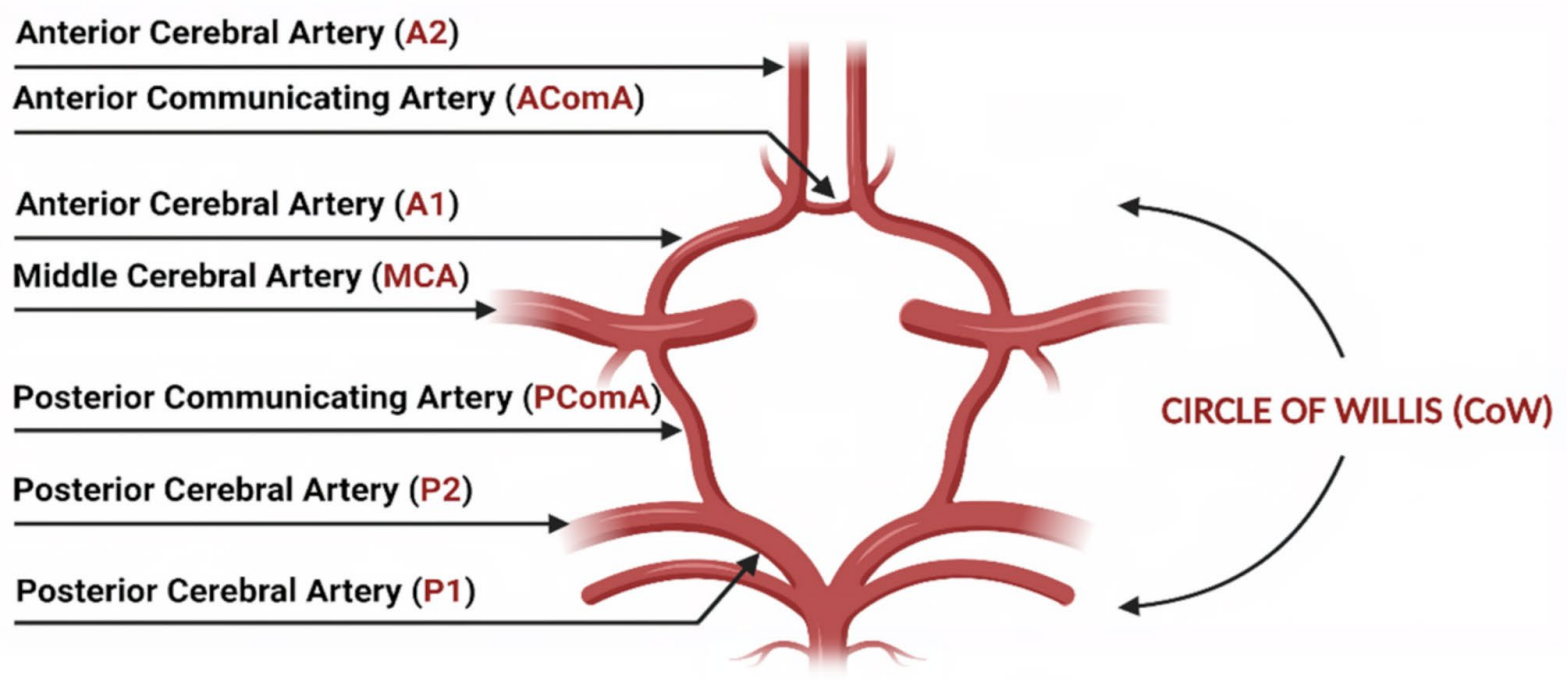
CoW anatomy with segment names and abbreviations. A complete circle includes the following segments: AcomA, A1 segments, PComAs, and P1 segments. MCAs are included in the diagram for anatomical completeness, although they are not considered structurally part of the CoW (Image created via BioRender.com)

One particular structure potentially impacted by such vascular changes is the Circle of Willis (CoW). The CoW is a key arterial ring located at the base of the brain that plays a vital role in the distribution and flow of cerebral blood, and in the maintenance of an adequate blood supply in case of arterial occlusion [10, 11]. Overall, a complete circle is characterised by a distinctive, symmetrical polygonal shape, consisting of interconnected segments of the anterior and posterior cerebral arteries, along with the anterior communicating artery and the posterior communicating arteries (Fig. 1) [12]. However, modern imaging techniques, in conjunction with autopsies, have revealed that only a relatively small portion of the population exhibits what is considered a “complete and non-variant” CoW configuration [13].

The variability in CoW anatomy may have notable physiological implications and can modify haemodynamics. Studies have shown that anatomical variations affecting the anterior cerebral arteries and the posterior communicating arteries play a crucial part in the development and potential rupture of cerebral aneurysms [14, 15]. Circles with absent segments have been, in addition, associated with the presence of various covert vascular brain injuries, including white matter disease, cerebral microbleeds, and the development of ischemic stroke [16–18]. Notably, Lin et al. found that, in relation to stroke outcomes, incomplete CoWs are often linked to a poorer prognosis [19].

The prevalence of anatomical variations in the CoW is widely reported in the literature, ranging from 53.9 to 82.5% [13]. The most frequently recorded variants include vessel hypoplasia, absence, and the presence of accessory segments [20]. Different classification systems for the characterisation of CoW variations have been used in the past, with great variability in the results [13, 21, 22]. Ayre et al. recently proposed a new comprehensive classification system for CoW anatomical variations with the aim of increasing the overall consistency of nomenclature and terminologies. Their classification includes criteria for standardising the definition of a “normal” circle, such as the presence of all segments including the anterior communicating artery (AComA), A1 segments of the anterior cerebral arteries (ACAs), posterior communicating arteries (PComAs), and P1 segments of the posterior cerebral arteries (PCAs) (Fig. 1), all segments originating from their natural origins, the absence of accessory arteries and an external diameter of > 1 mm for all CoW vessels [20].

Previous literature has explored the variability of the CoW in healthy populations [11, 21, 23], in stroke patients [10, 19, 24], or in individuals with other cerebrovascular diseases [14, 15], relying mainly on TOF MRA or autopsy data. To our knowledge, only one previous study has investigated the impact of prematurity on different CoW configurations, and it predominantly focused on preterm infants [25]. The aim of our current study was to map out the incidence of CoW anatomical variations in young adults born preterm, using a standardised pipeline and classification system with clear definitions of CoW completeness. We employed state-of-the-art imaging technology and the latest variant classifications to explore the potential impact of prematurity on vascular phenotypes in the CoW and discuss the physiological implications for the respective population.

## Methods

### Study design, governance and participants

Data for this research were obtained from two studies conducted at the Oxford Cardiovascular Clinical Research Facility and Oxford Centre of Clinical Magnetic Resonance Research, John Radcliffe Hospital, Division of Cardiovascular Medicine, University of Oxford, United Kingdom.

The first study, referred to as “Young Adult Cardiovascular Health Study (YACHT)”, was a cross-sectional observational study recruiting young adults aged 18 to 40 years with various cardiovascular risk factors, including hypertension and novel factors such as gestational age at birth. A total of 125 participants underwent MRI brain examinations, including 3D TOF MRA. Ethical approval was obtained from the South-Central Berkshire Research Ethics Committee (14/SC/0275). The study was registered on ClinicalTrials.gov (NCT02103231). Informed consent was obtained prior to study enrolment [26].

The second study, referred to as “Trial of Exercise to Prevent Hypertension in Young Adults (TEPHRA)” was a randomised controlled trial comprising young adults aged 18 to 35 years, including a subgroup born prematurely (less than 37 weeks gestation), with an awake ambulatory systolic and/or diastolic blood pressure between 115/75 mmHg and 150/95 mmHg, who had never been, nor were currently, medicated for hypertension. A total of 135 individuals underwent MRI brain examinations, in addition to 3D TOF MRA, with the full-term group having the scans repeated at two different timepoints. Informed consent was sought prior to enrolment in the trial. The study received approval from the University of Oxford as the host institution and the South-Central Research Ethics Committee (REC) for the National Health Service Health Research Authority (NHS HRA) (Reference 16/SC/0016) and it was registered on ClinicalTrials.gov (NCT02723552) [27].

### MR imaging data acquisition

Participants in both studies were scanned using a 3.0T MR scanner (Trio, A Tim, Siemens Healthineers AG, Erlangen, Germany) with a dedicated high-resolution 3D TOF MRA protocol with TR/TE = 23/8 ms, flip angle = 10°, FOV = 300 mm and voxel size = 0.6 × 0.6 × 0.6 mm. The TOF sequence was utilised to visualise the cerebral vasculature without the need for contrast agents’ injection [26, 27].

### Image curation and analysis

A standardised qualitative assessment of angiographic images was conducted. Of the original combined cohorts (*N* = 362), 107 3D TOF MRA datasets were excluded from further analysis for various reasons: 93 were duplications of the same participants, 10 were affected by motion artefacts, limiting the interpretation of CoW anatomy, 2 were characterised by insufficient anatomical coverage, with a significant portion of CoW anatomy not included in the native acquisition, and another 2 were datasets of participants with missing birth history data. These exclusions led to a total of 255 datasets being eligible for further analysis. Of these, there were 108 datasets of preterm and 147 of full-term young adults.

**Fig. 2:**
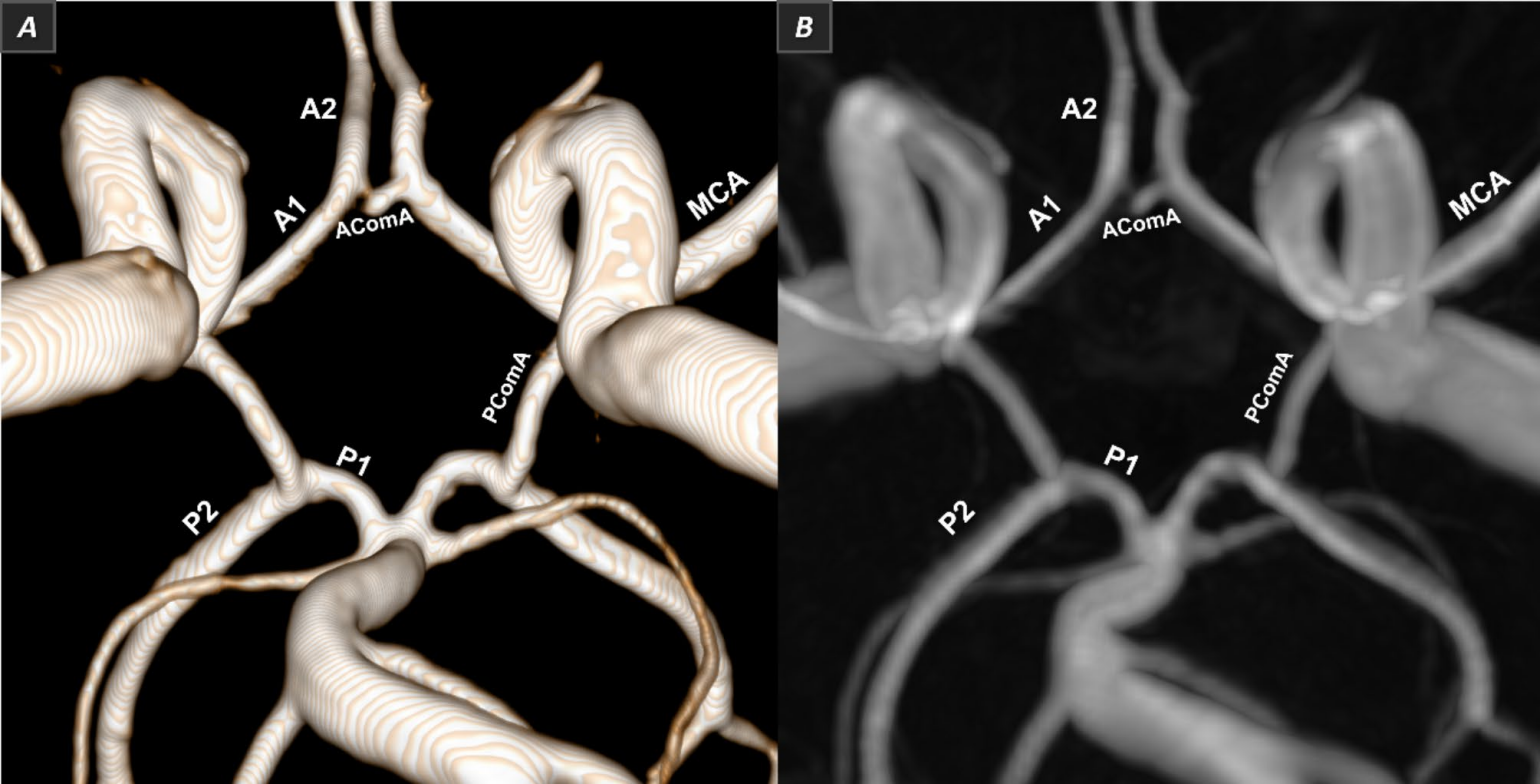
CoW anatomy with segment abbreviations. 3D CoW representation, along with its component vessels, displayed using both VR (image **A**) and MIP reconstructions (image **B**) in 3D Slicer (v5.6.1) (screenshot taken from native data)

The 3D TOF MRA datasets were evaluated by three observers blinded to the demographic and clinical details of the participants. The first observer is a lead MR radiographer with 10 + years of experience in neuroimaging, the second observer is a consultant neuroradiologist with 20 + years of experience in neuroimaging, and the third observer is an academic radiographer with 20 + years of experience in MRI/MRA image analysis. All observers used 3D Slicer (v5.6.1) (v5.6.1) [28]. The software displays 3D TOF images in their native acquired plane, as well as volume rendering (VR) or maximum intensity projection (MIP) (Fig. 2), enabling automatic multiplanar reconstruction (MPR) of the native plane (axial) into different spatial planes (coronal and sagittal). The entire study sample was analysed by the first observer, while a random subset of 20% and 10% of the cases was reviewed by the second and third observers, respectively, to ascertain reproducibility and consistency. Cohen’s kappa coefficient was calculated as a measure of inter-rater reliability [29].

**Fig. 3:**
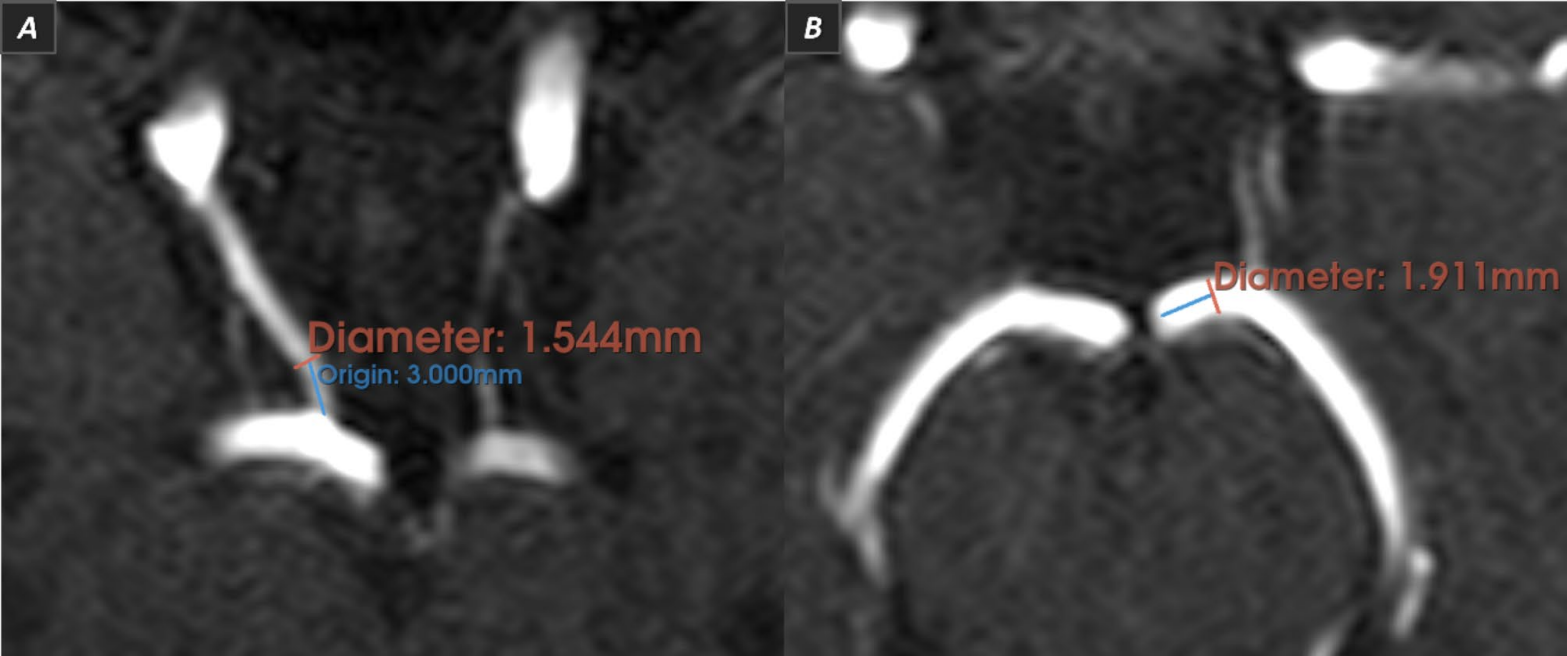
Lumen diameter measurement tool. The diameters of the right PComA (Image **A**) and left P1 (Image **B**) are measured through the axial / native plane of a 3D TOF MRA dataset

The size of each CoW segment was measured through the use of a lumen diameter measurement tool available on 3D Slicer, following the method originally established in Shatri et al. [30], and later revisited in Kızılgöz et al. [22]; hence, the vessel’s lumen was measured on the axial plane, 3 mm from the vessel origin, perpendicular to the elongation of the artery from the inner walls (Fig. 3).

In accordance to previous practice, when a CoW segment was too short, the middle part of the vessel was taken instead as a reference for measurement [22]. All the measurements were performed using the 3D slicer “default / recommended” window settings to avoid potential windowing biases that could lead to inconsistency in vessel analysis.

If an artery was not visualised on 3D MIP and/or VR, its presence was still investigated through the native source images to avoid 3D reconstruction biases for smaller vessels. Assessment of the native images was also done to ensure effective distinction between the PComAs and the anterior choroidal arteries, as well as potential overlapping pericallosal branches of the ACA. This involved a methodical slice-by-slice review of each axial section and careful observation of the distinct pathways characterising the arteries in sequence. Identifying the communication between the PComA and PCA was considered critical for determining the vessel’s configuration. A similar method was also applied to ensure appropriate differentiation of the PCAs from the superior cerebellar arteries.

### Definitions

An artery was considered “hypoplastic” if the lumen diameter measured was inferior to 1 mm, as per criteria established in Ayre et al. [20]. Arteries without any segmental visualisation or non-continuous segments on the native axial plane and related MPRs were labelled instead as “absent”. A CoW was defined as “variant” when at least one anatomical variation was present, using the new classification system introduced in Ayre et al. as a reference [20]. In the opposite case, the circle was labelled as “non-variant”. A CoW was defined as “complete” when all the vascular segments were present, irrespective of whether these segments were duplicated, hypoplastic, or of other types. In contrast, if any segment was identified as absent, the CoW was considered “incomplete”.

### Classification and differentiation of CoW anatomical variations and circle completeness

For each variant circle, the CoW configuration was assessed according to five main groups of CoW with anatomical variations [20]. Group 1 variant: Circles with one or more hypoplastic segments only. Group 2 variant: Circles with one or more absent segments only. Group 3 variant: Circles with hypoplastic and absent segments only. Group 4 variant: Circles with one or more accessory segments. Group 5 variant: Circles with other types of anatomical variation [20]. Additional file 1: Fig. S1 presents visual examples of 3D TOF MRA dataset reconstructions, illustrating the methodology involved for grouping different anatomical variations (group 1,2, and 3).

In our study a further distinction was made by establishing subgroups within group 4 and group 5. Group 4a variant: Circles with one or more accessory segments and no absent segment. Group 4b variant: Circles with one or more accessory segments and one or more absent segments (Additional file 2: Fig. S2). Group 5a variant: Circles with other types of anatomical variations and no absent segment. Group 5b variant: Circles with other types of anatomical variations and one or more absent segments (Additional file 3: Fig. S3). This additional differentiation was adopted to better define CoW completeness, distinguishing between “complete with variants” and “incomplete” circles. CoWs classified under Group 1, 4a, or 5a were defined as “complete with variants” since all vascular segments were considered present, even if some were hypoplastic, accessory, or other types. Circles classified under Group 2, 3, 4b, or 5b were labelled instead as “incomplete” due to the absence of one or more segments.

For each dataset analysed, a specific sample identifier and the study name were provided. A detailed description of the anatomical variations along with a variation code were noted, supported also by comments explaining the rationale behind each classification decision. Additional file 4: Table S1 illustrates schematically the “process” adopted to classify and differentiate CoW anatomical variations and establish circle completeness or incompleteness.

### Statistical analysis

Statistical analysis was performed using “R” (version 4.2.3). Key participant demographic characteristics, including sex, age, BMI, and gestational age, were summarised using means and standard deviations (SDs). Differences in the odds of various CoW variants between preterm (*N* = 108) and full-term (*N* = 147) populations were examined by employing multinomial logistic regression for the seven groups of anatomical variations. Binomial logistic regression was used, instead, to compare the odds between ‘complete and non-variant CoW’ and ‘CoW with variants”. An interaction term was included in the analysis to evaluate interaction effects between preterm birth status and sex. A *p*-value inferior to 0.05 was considered to indicate statistical significance.

## Results

### Participant characteristics

The results of the various participant demographics are shown below in Table 1.

**Table 1:** Participants’ characteristics for each category, distinguishing between sex (M = male), age, BMI, and gestational age

	Full-term *N* = 147	Pre-term *N* = 108
Sex M, n(%)	79 (54)	47 (45)
Age, M(SD)	26.3 (4.7)	26.5 (5.3)
BMI, M(SD)	24.2 (3.8)	23.5 (5.2)
Gestational Age, M(SD)	39.6 (1.4)	32.1 (3.2)

### Circle completeness and prevalence of anatomical variations

Out of 255 3D TOF MRA datasets analysed, 186 (72.94%) displayed a complete CoW. Among these, 91 datasets were categorised as “complete and non-variant,” while 95 were classified as “complete with variants” due to the occurrence of anatomical variations such as hypoplasia, accessory segments, or other variant types, excluding absent segments. In contrast, a total of 69 participants (27.06%) of the 3D TOF MRA datasets exhibited an incomplete circle, displaying at least one absent CoW segment. Table 2 provides a detailed breakdown of the different variant groups in relation to CoW completeness.

**Table 2:** Breakdown of different variant groups in relation to CoW completeness. A separate column displaying the number of excluded datasets is also included in the table for reference. Variant incidence is calculated based on the total sample of participants (*N* = 255)

Complete and Non-Variant CoW	CoW with Variants							Excluded Dataset
	Complete CoW with Variants			Incomplete CoW				
	Group 1 Variant Circles with one or more hypoplastic segments only	Group 4a Variant Circles with one or more accessory segments and no absent segment	Group 5a Variant Circles with other types of anatomical variation and no absent segment	Group 2 Variant Circles with one or more absent segments only	Group 3 Variant Circles with hypoplastic and absent segments only	Group 4b Variant Circles with one or more accessory segments and one or more absent segments	Group 5b Variant Circles with other types of anatomical variation and one or more absent segments	
91	46	39	10	40	12	15	2	107
35,69%	18.04%	15.29%	3.92%	15.69%	4.71%	5.88%	0.78%	
35,69% (Non-Variant)	**64**,**31% (With Variants)**							
72,94% (Complete)				**27**,**06% (Incomplete)**				

The overall prevalence of “CoW with variants” accounted for 164 cases (64.31%) of the entire sample. Within this data, a total of 219 variant segments were identified, revealing a broad range of anatomical variations.

Unilateral hypoplasia and unilateral absence were the most common anatomical variations out of all the variant segments (*N* = 219), detected in 66 (30%) and 64 (29%) individuals, respectively. These variations were most frequently observed in the PComAs. Triplication and duplication were also noted in 34 (16%) and 24 (11%) cases, particularly in the AComA and ACAs. Bilateral hypoplasia and bilateral absence were less frequent, accounting for 13 (6%) and 9 (4%) participants, respectively, and predominantly affected the PComAs. Unique variations categorised as “Other Types” were also less common (*N* = 11), including V-shaped configurations, segment elongation, and fenestration, with the most common affecting the AComA.

In general, the PComAs exhibited the most frequent variations, accounting for over 50% of the total CoW variants (*N* = 111). Unilateral PComA hypoplasia was observed in 49 cases and bilaterally in 13 cases. Unilateral PComA absence was found in 38 cases, and bilateral absence in 8 cases. AComA variations accounted for approximately one-fifth of the total (*N* = 47), with duplications (*N* = 18) being considerably more frequent than other types of variants affecting this segment. ACA variations were also observed in 33 individuals (15%), with ACA triplication being the most common (*N* = 32). A1 segments of the ACAs and P1 segments of the PCAs displayed fewer variations, accounting for 11 (5%) and 15 (7%) cases of the total, respectively. Notably, 30 variant datasets did not have a “variation code” available in the classification system introduced by Ayre et al. [20]. Table 3 summarises the frequency of CoW segments identified as “variant” and the types of anatomical variation.

**Table 3:** Frequency of CoW variants, distinguishing between type of anatomical variation and CoW segment affected

CoW Segment	Anatomical Variation							Total (*N*)	Total (%)
	Unilateral Hypoplasia	Unilateral Absence	Bilateral Hypoplasia	Bilateral Absence	Duplication	Triplication	Other Types*		
Anterior Communicating Artery (AComA)	12	10	0	0	18	2	5	**47**	**21%**
Anterior Cerebral Arteries (ACAs)	0	0	0	0	0	32	1	**33**	**15%**
A1 Segments ONLY of the ACAs	1	6	0	0	2	0	2	**11**	**5%**
Posterior Communicating Arteries (PComAs)	49	38	13	8	0	0	3	**111**	**51%**
Posterior Cerebral Arteries (PCAs)	0	0	0	0	2	0	0	**2**	**1%**
P1 Segments ONLY of the PCAs	4	10	0	1	0	0	0	**15**	**7%**
**Total (N)**	**66**	**64**	**13**	**9**	**22**	**34**	**11**	**219**	
**Total (%)**	**30%**	**29%**	**6%**	**4%**	**10%**	**16%**	**5%**		**100%**

### Inter-rater agreement

The Cohen’s kappa statistic for inter-rater agreement was 0.73 between the first and second observer, and 0.84 between the first and third observer. According to conservative criteria, values of 0.61–0.80 indicate “substantial agreement”, while values of 0.81–1.00 denote “almost perfect agreement” [31]. These results suggest an overall high degree of consistency between the first observer and the two additional observers in this study.

### Relationships between CoW variants, prematurity and sex

The frequency of CoW variants and CoW completeness in relation to full-term and preterm populations is presented below in Table 4.

**Table 4:** Frequency of CoW variants and CoW completeness in relation to full-term and preterm populations

	Full-Term *N* = 147		Pre-Term *N* = 108	
**CoW Variant Group**	*N*	%	*N*	%
Non-Variant	58	39	33	31
1	22	15	24	22
2	28	19	12	11
3	7	5	5	5
4a	19	13	20	19
4b	8	5	7	6
5a	4	3	6	6
5b	1	1	1	1
**CoW Completeness**	*N*	%	*N*	%
Complete and Non-Variant	58	39	33	31
Complete with Variants	45	31	50	46
Incomplete	44	30	25	23

**Fig. 4:**
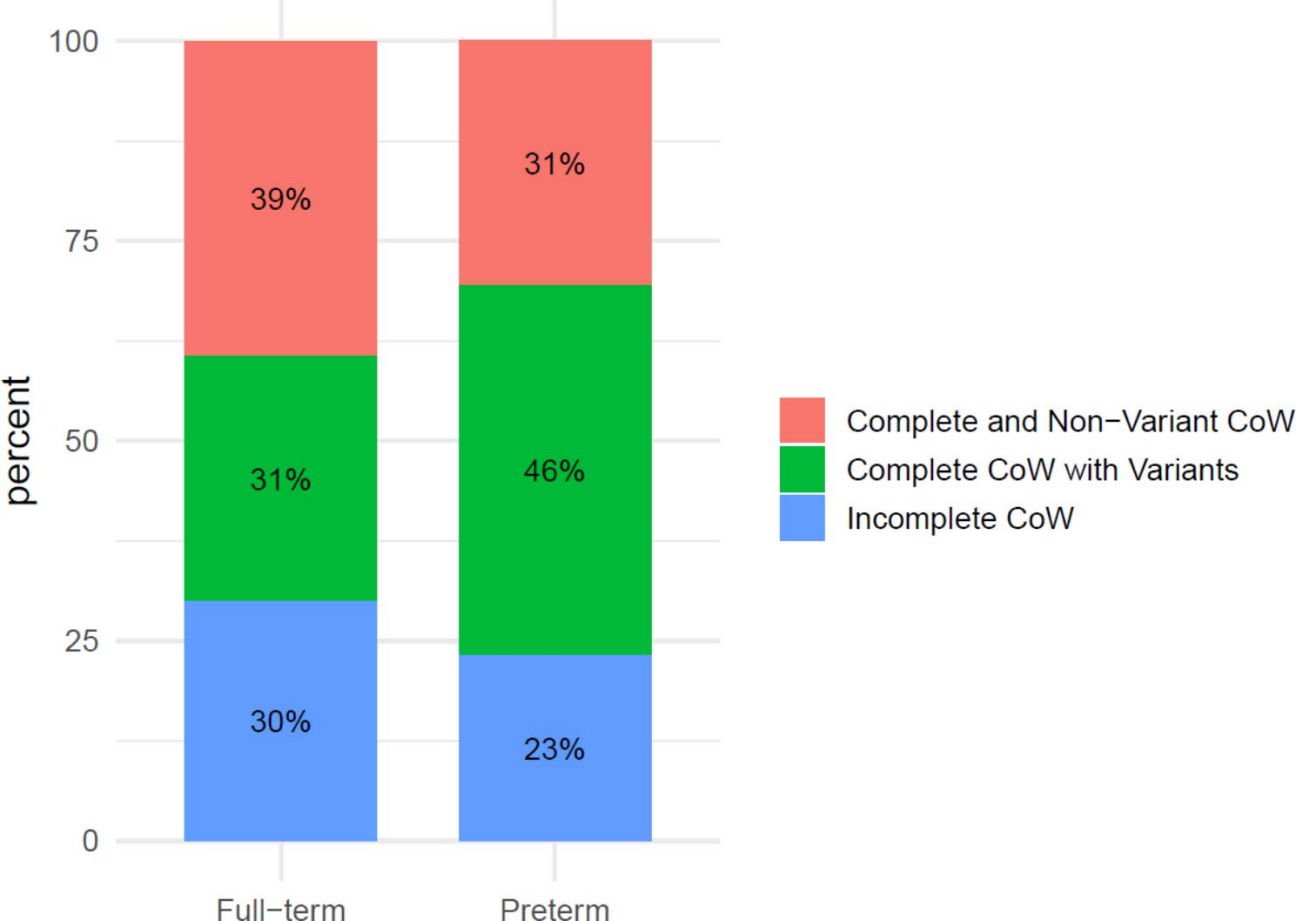
Proportion of CoW completeness in relation to full-term and preterm populations

The preterm population demonstrated significantly higher odds of having a complete CoW with variants compared to the full-term group. This result is statistically significant (*p* = 0.025) (Fig. 4, Additional file 4: Table S2).

**Fig. 5:**
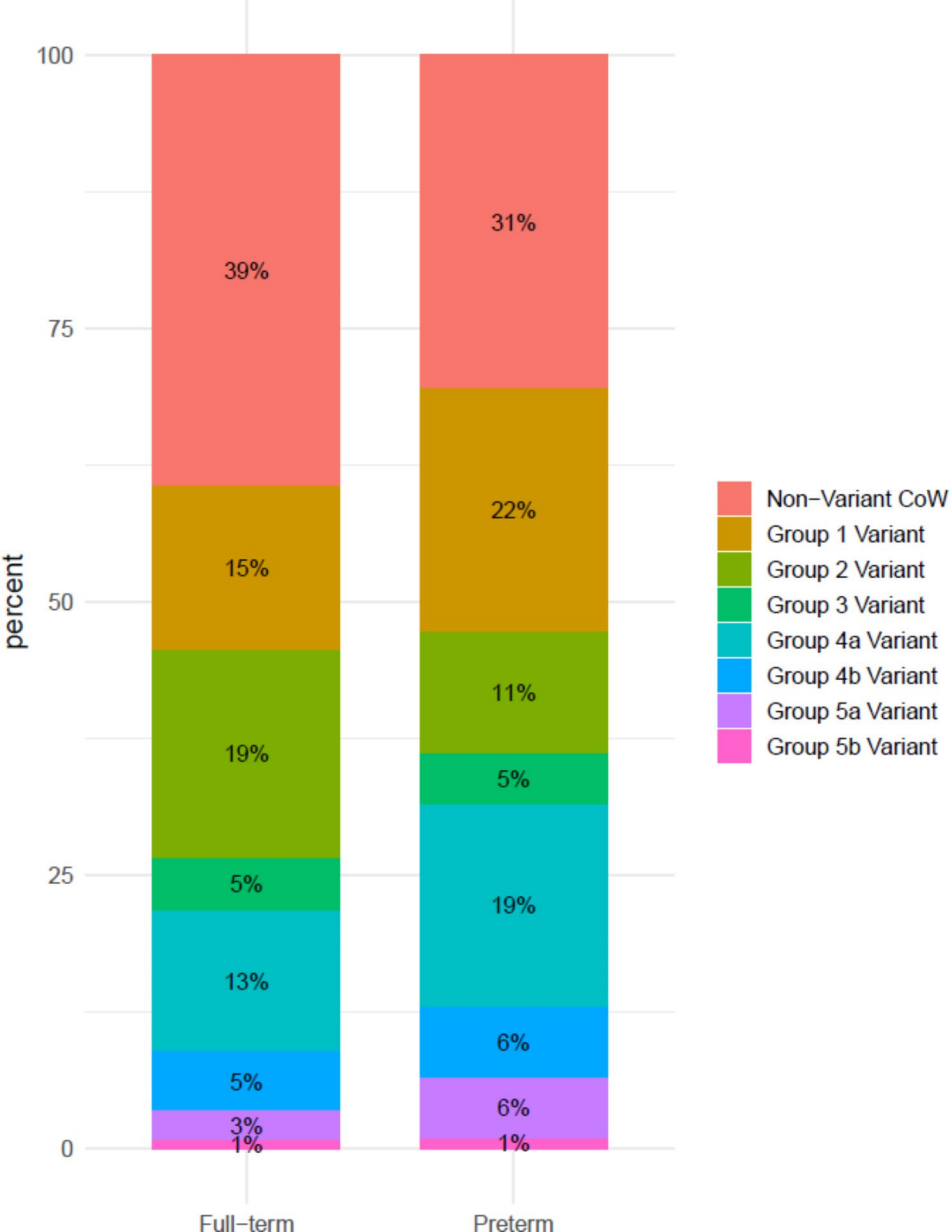
Proportion of CoW variants in relation to full-term and preterm populations

In addition, the odds ratio of the preterm group having a CoW group 1 variant (circles with one or more hypoplastic segments only) was found to be 1.9, nearly doubling that of the full-term population for displaying a similar CoW configuration. Nevertheless, this result did not reach statistical significance (*p* = 0.076) (Fig. 5, Additional file 4: Table S3).

Regarding the relationship between the prevalence of CoW variants and sex or degree of prematurity, male individuals born preterm were observed to have higher odds of displaying a CoW group 1 variant compared to the full-term male group (*p* = 0.024) (Additional file 4: Table S4). Preterm females, in contrast, were found to have statistically significantly higher odds of displaying a CoW group 4a variant (circles with one or more accessory segments, without any absent segments) compared to their full-term counterparts (*p* = 0.020) (Additional file 4: Table S5). When comparing individuals born preterm at < 32 weeks with those born at 32–37 weeks’ gestation, there were no significant differences in the prevalence of CoW variants.

## Discussion

### Variant vs. non-variant phenotypes

Overall, the prevalence of anatomical variations detected in this 3D TOF MRA sample supports the results previously presented in Jones et al., whereby anatomical variations are present in the majority of the global population. The distribution of these variations indicates significant heterogeneity in CoW configuration; variant types of CoWs have been implicated in altered cerebral haemodynamic and the effectiveness of collateral circulation [13].

Anatomical variations affecting the PComA were the most frequent ones detected (more than half of the total CoW variants). This segment is known to play a protective role in cases of posterior circulation ischemia and, a greater incidence of stroke has been previously reported in individuals with bilateral PComA absence [24, 32]. This emphasises the clinical significance of PComA variations and the need for tailored neurovascular assessment, diagnostic protocols, risk stratification, and interventions for different patient groups. Additionally, many individuals in our study were found to have an accessory ACA. This latter variant segment has been previously associated with the potential development of AComA aneurysms [33]. Admittedly, studies focusing on the frequency of this variant type are limited in the literature, even though it is cautiously reported to range between 1.3% and 3% [34]. This incidence is lower than the frequency (15%) detected in our datasets, and, thus, in view of the potential implications that ACA triplication may have on the development of certain neurovascular conditions, further research is needed.

### Complete vs. incomplete configurations

Nearly three out of four participants were found to have a complete CoW, either non-variant or with variants. Interestingly, preterm individuals were more prone to display complete CoW configurations with variants compared to those born full-term. In addition, the odds of having a complete circle with one or more hypoplastic segments only were almost double in the preterm population. This could indicate a potential connection between prematurity and the presence of certain CoW patterns, with significant relevance from a neurovascular perspective. However, further research to confirm this with a larger prospective sample size is encouraged. Individuals with a complete circle are more likely to have effective collateral circulation and, therefore, may be at lower risk of developing neurovascular adverse events than those with incomplete circles [17]. In the context of ischemia, symptoms normally appear when the cerebral blood flow is halved, commonly during a transient ischemic attack, over which the communicating arteries, if present, tend to enlarge to preserve the necessary level of brain perfusion [35]. When the circle is incomplete, the probability of a positive outcome decreases due to the overall impaired collateral circulation [19]. Nevertheless, it is also important to note that if one or more hypoplastic segments are present, the effectiveness of the collateral circulation is strictly related to the degree of hypoplasia. In cases where hypoplasia is considered extensive, the collateral ability of the CoW could still be functionally compromised, potentially still leading to stroke and irreversible brain damage [35].

The relationship between circulatory system phenotype and functionality in relation to prematurity has already been discussed by different groups, mainly working on CoW configuration and cardiac remodelling [5, 25]. These studies reported that very preterm infants (inferior to 30 weeks’ gestational age) had a complete circle with fewer anatomical variations compared to the full-term born population, as part of vascular remodelling. This unique phenotype was thought to act as a compensatory mechanism for the most vulnerable populations to maintain adequate blood supply to their brain in cases of vascular accidents [25]. Our study, carried out on young adults, suggests perhaps a different trend; our datasets of preterm born were, in fact, more frequently associated with distinctive anatomical variations compared to those participants born full-term. In this context, differences can be observed according to sex; preterm males were found to be more prone to exhibit CoW configurations with one or more hypoplastic segments compared to males born full-term. Furthermore, preterm females were more likely to display circles with one or more accessory segments, without any absent vessels, compared to their full-term female counterparts. However, no significant differences in CoW variants were observed concerning the severity of prematurity.

Our results about CoW completeness suggest that the preterm population is more likely to exhibit anatomically complete CoW configurations with variants such as hypoplasia, duplication or triplication, but without any absent segment. Preterm individuals are known to be at higher risk of developing different vascular conditions such as low systemic blood flow, when infants [36], but they also develop more frequently than their full-term-born counterpart conditions such as retinopathy of prematurity, intraventricular haemorrhage, necrotising enterocolitis as infants, and demonstrated altered cardiac structure and function as young adults [37–40]. All these studies corroborate that vascular remodelling may be associated with preterm birth and prematurity leaves potentially a permanent signature upon vascular phenotypes.

### Methodological considerations

Our study employed terminologies that might differ from those used in other studies, which may explain some discrepancies in results. Our definitions were consistently applied across all image processing and evaluations within our project. The term “complete” has been used interchangeably in previous literature to refer either to anatomical integrity or, in other texts, to denote the absence of variants [21, 41]. The inconsistent terminology used in different studies often makes it challenging to compare them fairly or directly. It is essential to understand what each definition represents to make clinical comparisons effectively. This variability in methodologies could complicate the generalisability of our findings.

It is also highly possible that the way a study defines a vessel as “hypoplastic” differs from the approach that is used by another. For example, our methodology was based on definition of “hypoplastic vessel” as a CoW segment with a diameter < 1 mm. In contrast, other studies have used a threshold < 0.8 mm [22, 23, 41], therefore automatically excluding those segments with diameters ranging from 0.8 to 1 mm, which our study considered instead hypoplastic. This variability in study approaches can potentially falsely reduce the overall reported prevalence of hypoplasia. Moreover, in the existing literature, studies often relied on the term “foetal type origin” in order to indicate the presence of a variant in the posterior compartment of the CoW [22, 23, 32]. This terminology was, however, not utilised in the most recent classification system [20], leading to a potential discrepancy in the existing reported prevalence.

Most studies investigating CoW anatomical variations, to date, have relied on TOF MRA data at different magnetic field strengths. Notably, superior signal-to-noise ratio (SNR) can be achieved by performing the TOF MRA sequence at 3.0T rather than at 1.5T [42] which could have implications on the resulting image quality, and consequently on vessel visualisation and CoW variation detection. In addition, when acquired at 1.5T, the TOF sequence performance has been increasingly associated with a “suboptimal” delineation of the AComA and PComA as a result of the slower or more turbulent flow within those vessels [10]. Our TOF MRA datasets were originally acquired with a 3.0T MR machine, where more robust imaging, with higher SNR, is achieved.

Some combinations of anatomical variations encountered in our study were not represented in the new classification system we used. For instance, while individual variation codes for a duplicated AComA and a hypoplastic P1 segment were available in Ayre et al. as “ACOMAD” and “P1H” respectively, the specific combination of these variations “ACOMAD-P1H” was not documented. To address this gap, each of these combinations was assigned a new code based on the nomenclature previously utilised in Ayre et al. Additional file 5: Table S6 summarises the new codes we proposed.

### Limitations

Despite the overall high inter-rater reliability found between observers, a certain level of disagreement arose in regards to the definition of a non-continuous segment as “absent” or “hypoplastic”. Similar to other studies investigating the prevalence of CoW anatomical variations [22, 23], our methods indicated as “absent” those vessels that were not connected to other arteries as per complete CoW textbook [20], regardless of their diameter. However, other authors [19, 21] suggested that in those cases the differentiation between “hypoplastic” and “absence” cannot be reliably performed as result of the limited resolution that characterised the TOF MRA sequence and the potential low flow rates in smaller arteries.

There are known limitations with using the TOF MRA technique due to its sensitivity to blood flow; thus, an absent vessel may simply have very slow-flowing blood inside it [21]. Contrast-Enhanced MRA (CE-MRA) could be a viable alternative to TOF MRA for assessing vessel presence and size, potential occlusion, and collateral status. Nevertheless, CE-MRA is often associated with lower spatial resolution compared to TOF MRA and requires the use of contrast media, which may not be suitable for imaging healthy volunteers. Therefore, TOF MRA remains a more appropriate choice for larger population studies like ours [43]. Other techniques, such as cadaveric dissection, may have their own challenges too, such as the dehydration of specimens or the varying use of preservation agents (formalin, ethanol, or other methods), which could alter vessel elasticity, thereby impacting diameter measurements [44, 45]. Although no statistically significant differences were found between the results obtained from cadaveric dissection and live patient imaging studies [13], these technical limitations might still negatively affect the reliability of the reported prevalence data.

The identified differences in CoW configurations can vary not only in relation to prematurity and sex but also in relation to ethnicity, age, lifestyle diseases, and factors affecting blood flow [13, 41]. Our study’s sample was restricted to healthy young adults living in the United Kingdom, with a certain level of heterogeneity in risk factors. This should be carefully taken into consideration before generalising our findings to other studies investigating the prevalence of CoW anatomical variations and the nature of CoW completeness.

The limited cerebral perfusion imaging data available for our participants prevented correlation of findings with downstream perfusion demands, which could have provided additional insight into the collateralisation patterns of the CoW. Furthermore, rare anatomical variations, such as primitive carotid-vertebrobasilar anastomoses, were not investigated in this study as they were not coded in the classification system we utilised. This underlines the importance of future work aimed at expanding this classification system.

## Conclusions

This study systematically evaluated anatomical variations in the Circle of Willis using high resolution 3D TOF MRA in a sample of 255 healthy young adults born preterm (*N* = 108) or full-term (*N* = 147). Our findings suggest the presence of distinctive patterns of CoW variants in preterm individuals, who are more likely to display anatomically complete circles but also have more variations, in particular hypoplastic segments for males and accessory segments for females. Furthermore, the lower likelihood of CoW configurations with absent segments in the preterm group, especially compared to circles characterised only by variants such hypoplasia, duplication and triplication, could potentially indicate the presence of an adaptive mechanism that preserves adequate cerebral perfusion for this vulnerable population. A distinct vascular phenotype of the CoW may be associated with premature birth which persists into young adulthood, highlighting therefore the need for specialised neurovascular assessments when prematurity is involved.

Our study introduces a reproducible and comprehensive imaging data analysis pipeline, but also underscores the need for extending the current classification system to less usual variant combinations and clarifying the terminology used, including but not limited to the definitions of “vessel hypoplasia” and “CoW completeness”. This would ensure an overall high level of consistency and generalisability in future research addressing this topic.

Finally, from a clinical perspective, our results pinpoint the necessity for further studies aimed at exploring the clinical implications of these variants across the cerebrovascular risk spectrum. We are rapidly approaching the first era with a relatively large preterm, middle-aged population. This fact, combined with our findings that CoW variants in this at-risk population often impact the PComA and ACA in ways that are associated with increased cerebrovascular risk, highlights the crucial and time-sensitive need to advance the work in this area.

## Electronic supplementary material

Below is the link to the electronic supplementary material.

Supplementary Material 1

Supplementary Material 2

Supplementary Material 3

Supplementary Material 4

Supplementary Material 5

## Data Availability

Data for this research were derived from two studies: the ‘Young Adult Cardiovascular Health Study (YACHT)’ (trial registration number: NCT02103231) and the ‘Trial of Exercise to Prevent Hypertension in Young Adults (TEPHRA)’ (trial registration number: NCT02723552). Ethical approvals for these studies were granted by the South-Central Berkshire Research Ethics Committee (14/SC/0275) and the University of Oxford as the host institution along with the South-Central Research Ethics Committee (Reference 16/SC/0016), respectively. It was the requirement of the trials that written informed consent was obtained prior to the enrolment of the participants. The investigators ensured that the two studies were conducted in accordance with the principles of the Declaration of Helsinki. The investigators ensured that the two studies were conducted in accordance with relevant regulations and Good Clinical Practice. This research is a secondary analysis of data from the “YACHT” and “TEPHRA” trials and does not require a separate clinical trial registration.

## Abbreviations

3D: Three–Dimensional
ACA: Anterior Cerebral Artery
AComA: Anterior Communicating Artery
BMI: Body Mass Index
CE: MRA–Contrast–Enhanced Magnetic Resonance Angiography
CoW: Circle of Willis
MCA: Middle Cerebral Artery
MRI: Magnetic Resonance Imaging
MRA: Magnetic Resonance Angiography
NHS HRA: National Health Service Health Research Authority
PCA: Posterior Cerebral Artery
PComA: Posterior Communicating Artery
REC: Research Ethics Committee
SD: Standard Deviation
SNR: Signal–To–Noise Ratio
TEPHRA: Trial of Exercise to Prevent Hypertension in Young Adults
TOF: Time–of–Flight
YACHT: Young Adult Cardiovascular Health Study
