# Correction: Distinct circle of willis anatomical configurations in healthy preterm born adults: a 3D time-of-flight magnetic resonance angiography study

**DOI:** 10.1186/s12880-025-01584-6

**Published:** 2025-02-17

**Authors:** Julien Greggio, Christina Malamateniou, Kelly Pegoretti Baruteau, Constantino Carlos Reyes-Aldasoro, Odaro J. Huckstep, Jane M. Francis, Wilby Williamson, Paul Leeson, Adam J. Lewandowski, Winok Lapidaire

**Affiliations:** 1 Department of Midwifery & Radiography, City St George’s, School of Health & Psychological Sciences, University of London, Clerkenwell Campus, London, EC1V 0HB UK; 2 Department of Neuroimaging, Kings’ College London, London, SE5 8AF UK; 3 Lysholm Department of Neuroradiology, National Hospital for Neurology and Neurosurgery, University College London Hospitals NHS Foundation Trust, London, WC1N 3BG UK; 4 Elizabeth Garrett Anderson, Institute for Women’s Health, University College London, London, UK; 5 Department of Computer Science, City St George’s, School of Health & Medical Sciences, University of London, London, EC1V 0HB UK; 6 Integrated Pathology Unit, Division of Molecular Pathology, The Institute of Cancer Research, Sutton, UK; 7 Department of Biology, United States Air Force Academy, Colorado Springs, CO USA; 8 MyCardium AI, Liverpool, UK; 9 School of Medicine, Trinity College Dublin, Dublin, Ireland; 10 Oxford Cardiovascular Clinical Research Facility, Division of Cardiovascular Medicine, Radcliffe Department of Medicine, Oxford Heart Centre, John Radcliffe Hospital, University of Oxford, Level 1, Oxford, OX3 9DU UK; 11 Department of Population Health, University of Oxford, Oxford, OX3 7LF UK

Correction to: Greggio et al. BMC Medical Imaging (2025) 25:33.

10.1186/s12880-025-01562-y.

Following the publication of the original article, the authors reported that the funding statement was incorrect.

**Incorrect**:

This research received no specific grant from any funding agency in the public, commercial, or not-for-profit sectors.

**Correct**:

Supported by funding from the Wellcome Trust (105741/Z/14/Z), British Heart Foundation (FS/18/3/33292 and PG 13/58/30397), the Oxford BHF Centre for Research Excellence, and the National Institute for Health Research Oxford Biomedical Research Centre.

The original article has been corrected.

