# Supplementary material for: Distinct Circle of Willis anatomical configurations in healthy preterm born adults: a 3D time-of-flight magnetic resonance angiography study
Source: BMC Med Imaging. 2025 Jan 30;25:33. doi: 10.1186/s12880-025-01562-y (PMC11783829; doi:10.1186/s12880-025-01562-y)
Supplement: Supplementary file 1 — Supplementary Material 1 [file 12880_2025_1562_MOESM1_ESM.docx]

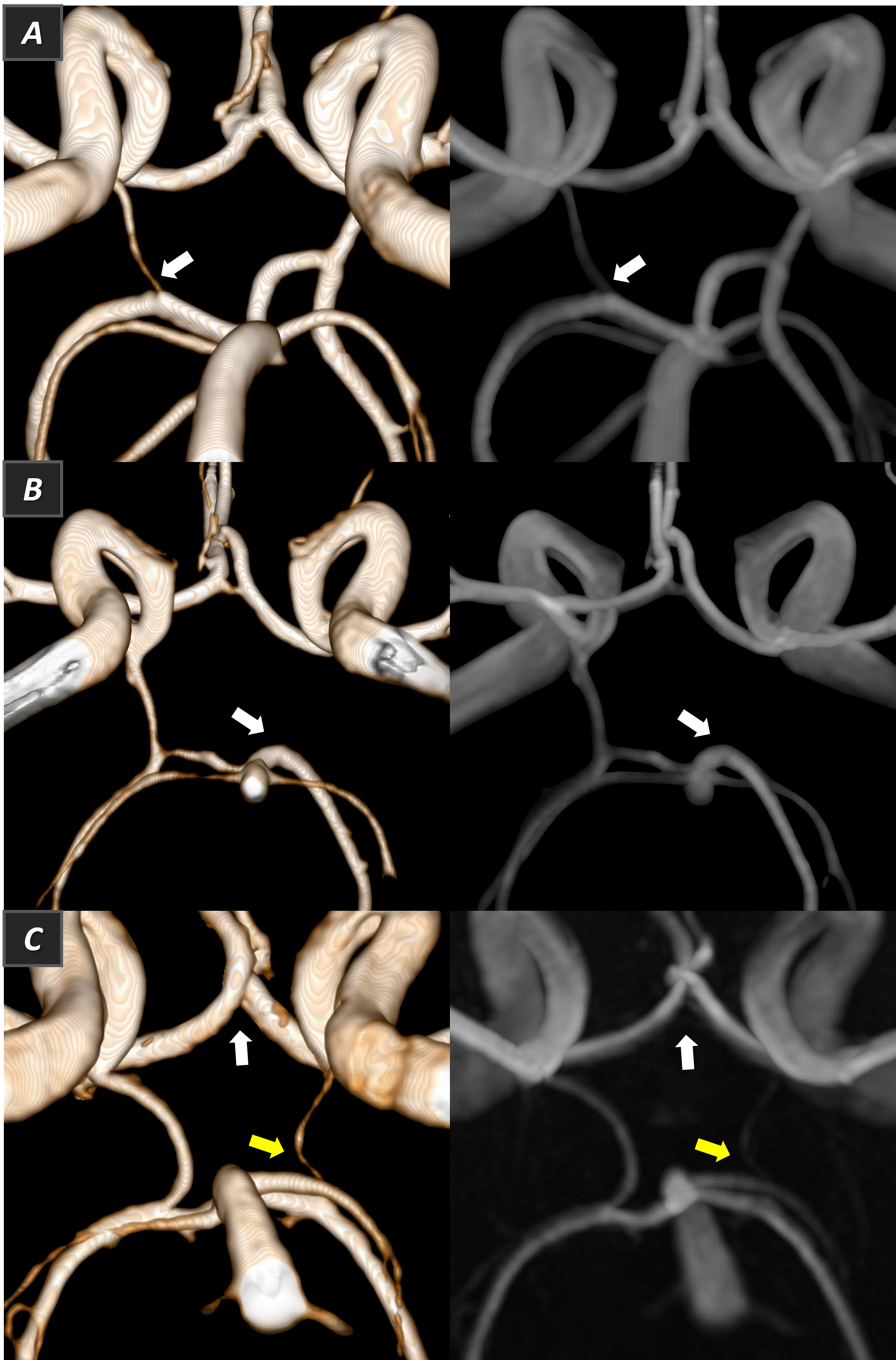


**Additional file 1 (.png): Fig. S1** Classification and differentiation of CoW variants (Group 1, Group 2 and Group 3). Image A, B and C showcase 3D TOF MRA VR and MIP reconstructions. In image A, the right PComA is hypoplastic (white arrow), with the circle therefore falling into group 1. In image B, the left PComA is absent (white arrow), thus placing the circle into group 2. In image C, the AComA is absent (white arrow) and the left PComA hypoplastic (yellow arrow), with the circle consequently categorised as group 3 (screenshot taken from native data).
