# Supplementary material for: Distinct Circle of Willis anatomical configurations in healthy preterm born adults: a 3D time-of-flight magnetic resonance angiography study
Source: BMC Med Imaging. 2025 Jan 30;25:33. doi: 10.1186/s12880-025-01562-y (PMC11783829; doi:10.1186/s12880-025-01562-y)
Supplement: Supplementary file 2 — Supplementary Material 2 [file 12880_2025_1562_MOESM2_ESM.docx]

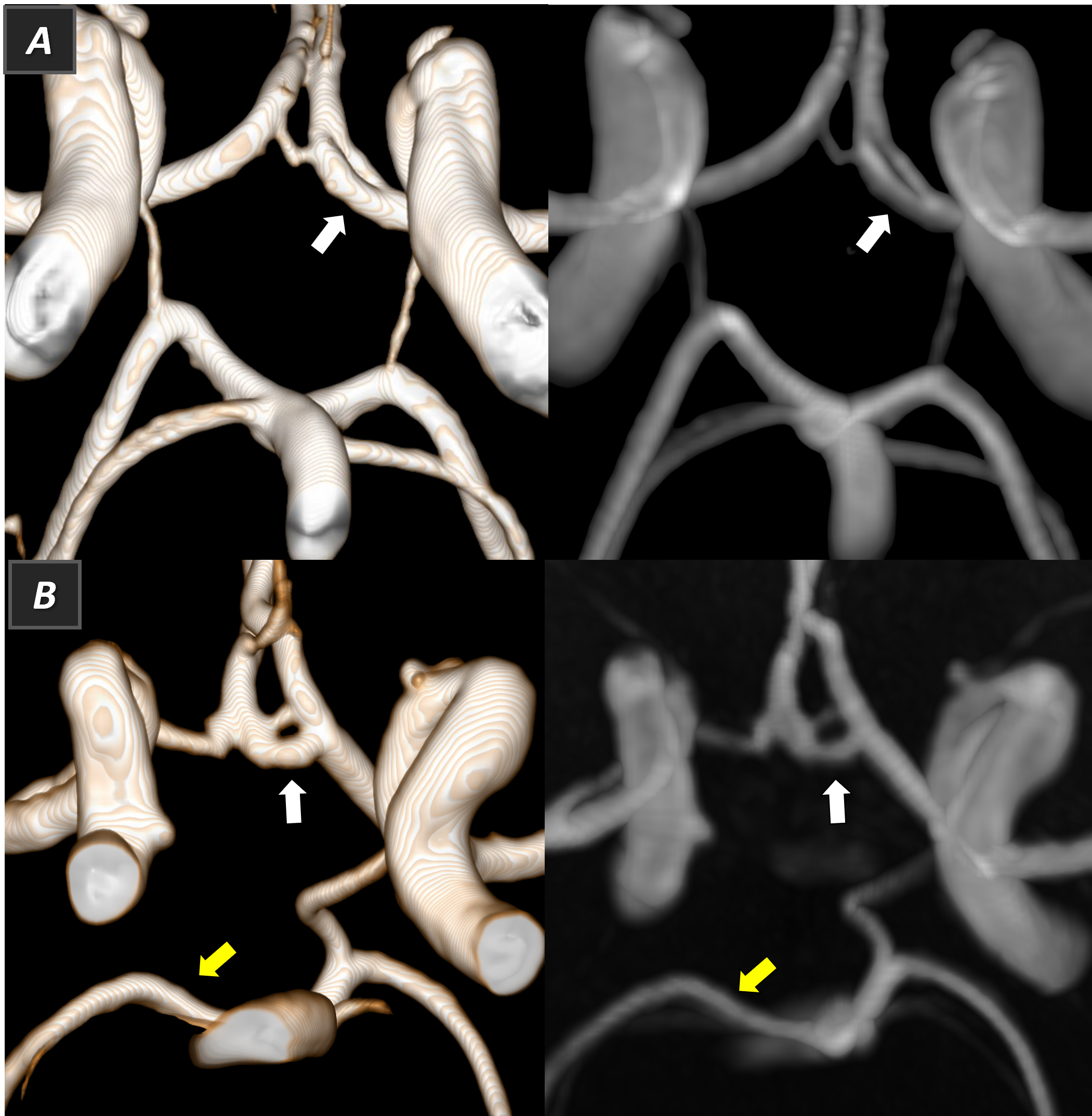


**Additional file 2 (.png): Fig. S2** Classification and differentiation of CoW variants (Group 4a and Group 4b). Image A and B display 3D TOF MRA VR and MIP reconstructions. In image A, the ACA is triplicated (white arrow), with a circle therefore falling into group 4a. In image B, the AComA is duplicated (white arrow) and the right PComA is absent (yellow arrow), thus the circle is categorised into group 4b (screenshot taken from native data).
