# Supplementary material for: Distinct Circle of Willis anatomical configurations in healthy preterm born adults: a 3D time-of-flight magnetic resonance angiography study
Source: BMC Med Imaging. 2025 Jan 30;25:33. doi: 10.1186/s12880-025-01562-y (PMC11783829; doi:10.1186/s12880-025-01562-y)
Supplement: Supplementary file 3 — Supplementary Material 3 [file 12880_2025_1562_MOESM3_ESM.docx]

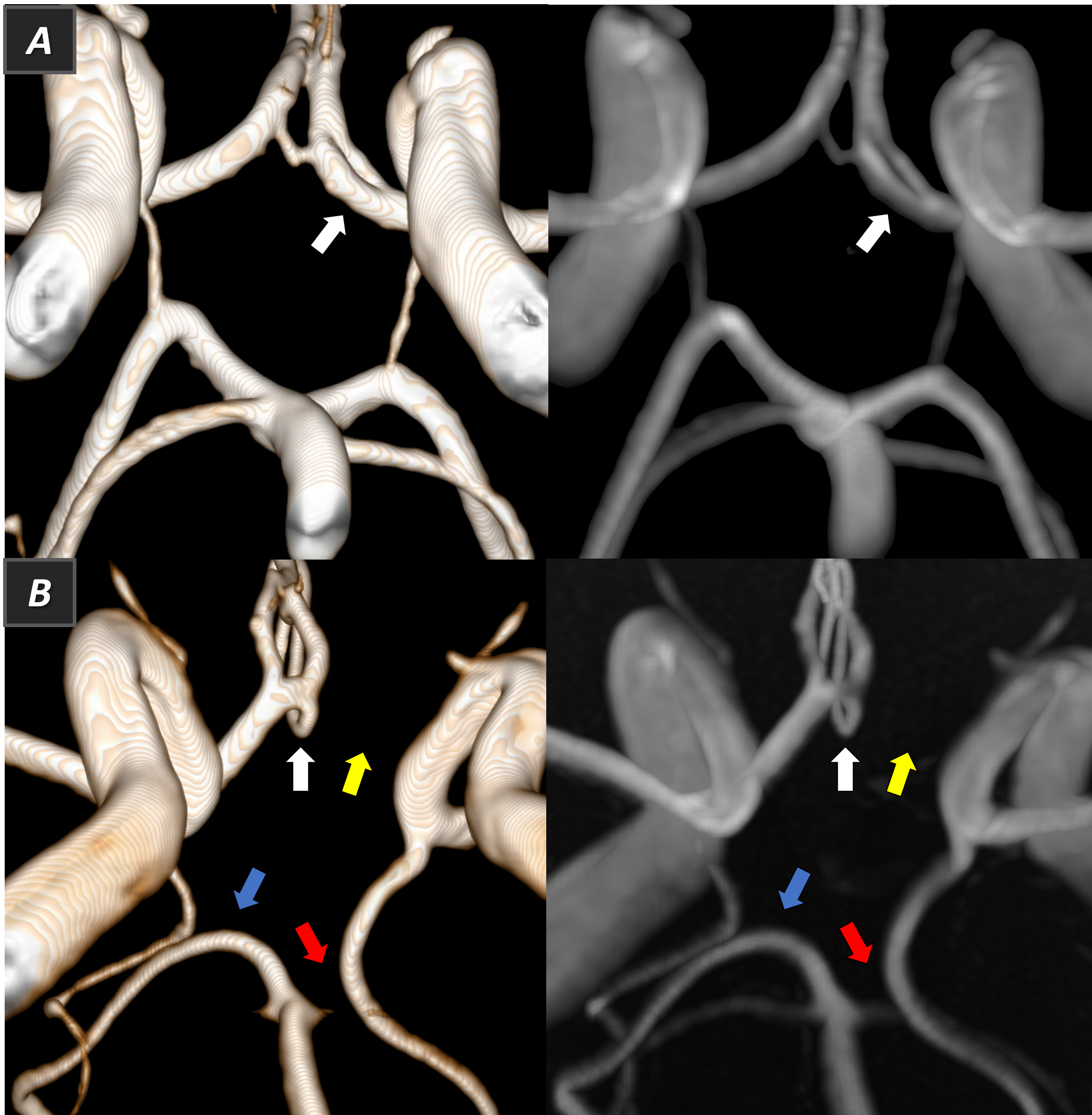


**Additional file 3 (.png): Fig. S3** Classification and differentiation of CoW variants (Group 5a and Group 5b). Image A and B display 3D TOF MRA VR and MIP reconstructions. In image A, the left A1 segment is fenestrated (white arrow), placing the circle into group 5a. In image B, the right A1 segment is fenestrated (white arrow), the AComA is absent (yellow arrow), the right PComA is absent (blue arrow) and the left PComA is absent (red arrow), with a circle consequently falling into group 5b (screenshot taken from native data).
