# Supplementary material for: Distinct Circle of Willis anatomical configurations in healthy preterm born adults: a 3D time-of-flight magnetic resonance angiography study
Source: BMC Med Imaging. 2025 Jan 30;25:33. doi: 10.1186/s12880-025-01562-y (PMC11783829; doi:10.1186/s12880-025-01562-y)
Supplement: Supplementary file 4 — Supplementary Material 4 [file 12880_2025_1562_MOESM4_ESM.docx]

| Sample Name | Study Name | Complete and Non-Variant CoW | CoW with Variants | | | | | | | Description of the Anatomical Variation (if present) | Variation Code | Comments |
| --- | --- | --- | --- | --- | --- | --- | --- | --- | --- | --- | --- | --- |
|  |  |  | **Complete CoW with Variants** | | | **Incomplete CoW** | | | |  |  |  |
|  |  |  | **Group 1 Variant** Circles with one or more hypoplastic segments only | **Group 4a Variant** Circles with one or more accessory segments and no absent segment | **Group 5a Variant** Circles with other types of anatomical variations and no absent segment | **Group 2 Variant** Circles with one or more absent segments only | **Group 3 Variant** Circles with hypoplastic and absent segments only | **Group 4b Variant** Circles with one or more accessory segments and one or more absent segments | **Group 5b Variant** Circles with other types of anatomical variations and one or more absent segments |  |  |  |
| YT*** | YACHT |  |  |  |  |  | **X** |  |  | Unilateral hypoplastic PComA and contralateral absent PComA | PCOMA^H^—^C^PCOMA^A^ | Left PComA is hypoplastic. Right PComA is a “non-continuous” segment and therefore will be considered absent |

**Additional file 4: Table S1** Process of CoW variant classification and differentiation related to circle completeness.

| **CoW Completeness** | **Estimate** | **Std Error** | **Statistic** | **p.value** | **expB** |
| --- | --- | --- | --- | --- | --- |
| **Complete with variants** | 0.669 | 0.299 | 2.233 | 0.025 | 1.952 |
| **Incomplete** | -0.001 | 0.332 | -0.004 | 0.996 | 0.998 |

**Additional file 4: Table S2** Statistical differences in CoW completeness: Full-term vs. Preterm.

| **Group Variant** | **Estimate** | **Std Error** | **Statistic** | **p.value** | **expB** |
| --- | --- | --- | --- | --- | --- |
| **1** | 0.650 | 0.366 | 1.773 | 0.076 | 1.917 |
| **2** | -0.283 | 0.408 | -0.694 | 0.487 | 0.753 |
| **3** | 0.227 | 0.624 | 0.364 | 0.715 | 1.255 |
| **4a** | 0.615 | 0.387 | 1.587 | 0.112 | 1.850 |
| **4b** | 0.430 | 0.561 | 0.766 | 0.443 | 1.537 |
| **5a** | 0.969 | 0.681 | 1.422 | 0.154 | 2.636 |
| **5b** | 0.564 | 1.430 | 0.394 | 0.693 | 1.758 |

**Additional file 4: Table S3** Statistical differences in CoW variant odds: Full-term vs. Preterm.

| **Group Variant** | **Estimate** | **Std Error** | **Statistic** | **p.value** | **expB** |
| --- | --- | --- | --- | --- | --- |
| **1** | 1.265 | 0.562 | 2.251 | 0.0243 | 3.545 |
| **2** | 0.656 | 0.575 | 1.142 | 0.253 | 1.928 |
| **3** | 0.875 | 0.773 | 1.131 | 0.257 | 2.400 |
| **4a** | 0.069 | 0.647 | 0.106 | 0.915 | 1.071 |
| **4b** | 0.693 | 0.751 | 0.922 | 0.356 | 2.000 |
| **5a** | 2.197 | 1.217 | 1.805 | 0.071 | 9.000 |
| **5b** | -9.919 | 246.863 | -0.040 | 0.967 | 0.00004 |

**Additional file 4: Table S4** Statistical differences in CoW variant odds: Full-term Males vs. Preterm Males

| **Group Variant** | **Estimate** | **Std Error** | **Statistic** | **p.value** | **expB** |
| --- | --- | --- | --- | --- | --- |
| **1** | 0.255 | 0.505 | 0.506 | 0.612 | 1.291 |
| **2** | -1.284 | 0.691 | -1.856 | 0.063 | 2.767 |
| **3** | -0.437 | 1.254 | -0.348 | 0.727 | 6.458 |
| **4a** | 1.354 | 0.583 | 2.320 | 0.020 | 3.874 |
| **4b** | 0.661 | 0.952 | 0.694 | 0.487 | 1.937 |
| **5a** | 0.255 | 0.860 | 0.297 | 0.766 | 1.291 |
| **5b** | 2.190 | 390.418 | 0.031 | 0.975 | 1.968 |

**Additional file 4: Table S5** Statistical differences in CoW variant odds: Full-term Females vs. Preterm Females
