# Supplementary material for: Distinct Circle of Willis anatomical configurations in healthy preterm born adults: a 3D time-of-flight magnetic resonance angiography study
Source: BMC Med Imaging. 2025 Jan 30;25:33. doi: 10.1186/s12880-025-01562-y (PMC11783829; doi:10.1186/s12880-025-01562-y)
Supplement: Supplementary file 5 — Supplementary Material 5 [file 12880_2025_1562_MOESM5_ESM.docx]

| **Study** | **Group** | **New Variation Code** | **Description of the variant circle** |
| --- | --- | --- | --- |
| YACHT | 4b | ACA^Tl^—P1^A^ | Lateral triplicated ACA and unilateral absent P1 |
| YACHT | 5a | ACA^Az^ | Azygous ACA |
| YACHT | 4a | ACOMA^D^—P1^H^ | Duplicated AComA and unilateral hypoplastic P1 |
| YACHT | 4b | ACA^Tm^—A1^A^—^B^PCOMA^H^ | Medial triplicated ACA, unilateral absent A1 and bilateral hypoplastic PComAs |
| YACHT | 3 | ACOMA^H^—PCOMA^H^—^C^P1^A^ | Hypoplastic AComA, unilateral hypoplastic PComA and contralateral absent P1 |
| YACHT | 4b | ACOMA^A^—A1^D^ | Absent AComA and unilateral duplicated A1 |
| YACHT | 3 | ACOMA^H^—PCOMA^A^ | Hypoplastic AComA and unilateral absent PComA |
| YACHT | 4b | ACA^Tm^—A1^A^—PCOMA^A^— ^C^P1^A^ | Medial triplicated ACA, unilateral absent A1, unilateral absent PComA and contralateral absent P1 |
| YACHT | 3 | ACOMA^A^—PCOMA^H^ | Absent AComA and unilateral hypoplastic PComA |
| YACHT | 5a | ACOMA^Vs^ | V-shaped AComA |
| YACHT | 4a | ACA^Tm^—ACOMA^D^—PCOMA^H^ | Medial triplicated ACA, duplicated AComA and unilateral hypoplastic PComA |
| YACHT | 5b | ACOMA^A^— A1^F^—PCOMA^H^ | Absent AComA, unilateral fenestrated A1 and unilateral hypoplastic PComA |
| YACHT | 3 | PCOMA^A^— ^C^P1^A^ | Unilateral absent PComA and contralateral absent P1 |
| TEPHRA | 5b | PCOMA^F^—^C^PCOMA^A^ | Unilateral fenestrated PComA and contralateral absent PComA |
| TEPHRA | 3 | PCOMA^A^— ^C^P1^A^ | Unilateral absent PComA and contralateral absent P1 |
| TEPHRA | 4b | ACA^Tm^—A1^A^ | Medical triplicated ACA and unilateral absent A1 |
| TEPHRA | 4b | ACA^Tm^—PCOMA^H^—^C^PCOMA^A^ | Medial triplicated ACA, unilateral hypoplastic PComA and contralateral absent PComA |
| TEPHRA | 4b | ACA^Tm^—^B^PCOMA^A^ | Medial triplicated ACA and bilateral absent PComAs |
| TEPHRA | 5a | A1^F^ | Fenestrated A1 |
| TEPHRA | 5a | ACOMA^Vs^ | V-shaped AComA |
| TEPHRA | 5a | ACOMA^Vs^ | V-shaped AComA |
| TEPHRA | 5a | A1^F^ | Fenestrated A1 |
| TEPHRA | 4b | ACOMA^D^—P1^A^ | Duplicated AComA and unilateral absent P1 |
| TEPHRA | 3 | PCOMA^A^— ^C^P1^A^ | Unilateral absent PComA and contralateral absent P1 |
| TEPHRA | 5a | PCOMA^E^ | Unilateral elongated PComA |
| TEPHRA | 5a | PCOMA^E^ | Unilateral elongated PComA |
| TEPHRA | 3 | ACOMA^A^—PCOMA^H^ | Absent AComA and unilateral hypoplastic PComA |
| TEPHRA | 4b | ACA^Tm^—ACOMA^D^—PCOMA^A^ | Medial triplicated ACA, duplicated AComA, and unilateral absent PComA |
| TEPHRA | 4b | ACOMA^T^—PCOMA^A^ | Triplicated AComA and unilateral absent PComA |
| TEPHRA | 4a | ACOMA^D^—P1^H^ | Duplicated AComA and unilateral hypoplastic P1 |

**Additional file 5 (.docx): Table S6** Summary of the new variation codes assigned to all the undocumented variants or combination of variants
